# A MALDI-TOF MS library for rapid identification of human commensal gut bacteria from the class *Clostridia*

**DOI:** 10.3389/fmicb.2023.1104707

**Published:** 2023-02-21

**Authors:** Paul Tetteh Asare, Chi-Hsien Lee, Vera Hürlimann, Youzheng Teo, Aline Cuénod, Nermin Akduman, Cordula Gekeler, Afrizal Afrizal, Myriam Corthesy, Claire Kohout, Vincent Thomas, Tomas de Wouters, Gilbert Greub, Thomas Clavel, Eric G. Pamer, Adrian Egli, Lisa Maier, Pascale Vonaesch

**Affiliations:** ^1^Department of Fundamental Microbiology, University of Lausanne, Lausanne, Switzerland; ^2^Department of Epidemiology and Public Health, Swiss Tropical and Public Health Institute, Basel, Switzerland; ^3^University of Basel, Basel, Switzerland; ^4^Applied Microbiology Research, Department of Biomedicine, University of Basel, Basel, Switzerland; ^5^Clinical Bacteriology and Mycology, University Hospital of Basel, Basel, Switzerland; ^6^Interfaculty Institute of Microbiology and Infection Medicine Tübingen, University of Tübingen, Tübingen, Germany; ^7^Cluster of Excellence ‘Controlling Microbes to Fight Infections’, University of Tübingen, Tübingen, Germany; ^8^Functional Microbiome Research Group, Institute of Medical Microbiology, RWTH University Hospital, Aachen, Germany; ^9^Institute of Microbiology of the University of Lausanne, University Hospital Centre (CHUV), Lausanne, Switzerland; ^10^Duchossois Family Institute, Division of Infectious Diseases and Global Health, University of Chicago, Chicago, IL, United States; ^11^BioAster, Paris, France; ^12^PharmaBiome AG, Schlieren, Switzerland

**Keywords:** human gut microbiota, *Clostridia*, MALDI-TOF MS, commensal bacteria, anaerobic, bacterial identification, culturomics, next-generation probiotics

## Abstract

**Introduction:**

Microbial isolates from culture can be identified using 16S or whole-genome sequencing which generates substantial costs and requires time and expertise. Protein fingerprinting *via* Matrix-assisted Laser Desorption Ionization–time of flight mass spectrometry (MALDI-TOF MS) is widely used for rapid bacterial identification in routine diagnostics but shows a poor performance and resolution on commensal bacteria due to currently limited database entries. The aim of this study was to develop a MALDI-TOF MS plugin database (CLOSTRI-TOF) allowing for rapid identification of non-pathogenic human commensal gastrointestinal bacteria.

**Methods:**

We constructed a database containing mass spectral profiles (MSP) from 142 bacterial strains representing 47 species and 21 genera within the class *Clostridia*. Each strain-specific MSP was constructed using >20 raw spectra measured on a microflex Biotyper system (Bruker-Daltonics) from two independent cultures.

**Results:**

For validation, we used 58 sequence-confirmed strains and the CLOSTRI-TOF database successfully identified 98 and 93% of the strains, respectively, in two independent laboratories. Next, we applied the database to 326 isolates from stool of healthy Swiss volunteers and identified 264 (82%) of all isolates (compared to 170 (52.1%) with the Bruker-Daltonics library alone), thus classifying 60% of the formerly unknown isolates.

**Discussion:**

We describe a new open-source MSP database for fast and accurate identification of the *Clostridia* class from the human gut microbiota. CLOSTRI-TOF expands the number of species which can be rapidly identified by MALDI-TOF MS.

## Introduction

*Clostridia* is an important class of Gram-positive, often strictly anaerobic, rod-shaped, spore-forming bacteria within the phylum *Bacillota* (formerly *Firmicutes*). Some *Clostridia* spp. are pathogenic in humans and animals such as *C. botulinum*, *C. tetani*, *and C. difficile*. However, most members of this class play an important role in the human intestinal tract, where they contribute, among others, to the production of the short-chain fatty acid butyrate (Beyer-Sehlmeyer et al., 2003; Paparo et al., 2017; Cruz-Morales et al., 2019).

Depletion of butyrate-producing *Clostridia* from the intestinal microbiota has been associated with multiple intra-and extraintestinal diseases such as, i.e., childhood stunting (Vonaesch et al., 2018), inflammatory bowel disease (Eeckhaut et al., 2013; Gevers et al., 2014), colorectal cancer (Wu et al., 2013), and cardiometabolic disease (Karlsson et al., 2012; Wang et al., 2012; Le Chatelier et al., 2013). Further, depletion in butyrate producers was shown to decrease colonization resistance to enteric pathogens (Rivera-Chávez et al., 2016). Overall, there is thus a clear link between disease and the absence of butyrate producers. Owing to their important role in health and disease, efforts have multiplied to isolate and characterize these bacteria and eventually use them as so-called next-generation probiotics (NGPs); bacteria directly isolated from healthy humans, that are re-introduced in diseased individuals as health-promoting strains (O’Toole et al., 2017; Chang et al., 2019; Lin et al., 2019). The identification and characterization of novel NGPs heavily rely on culture collections and thus on tools that can rapidly identify commensal bacteria.

The current gold standard to identify bacterial isolates are full-length 16S rRNA gene or whole-genome sequencing (WGS) (Browne et al., 2016; Lagkouvardos et al., 2019). WGS is time-consuming, costly and requires a specific expertise. For this reason, it is rarely used for species identification in clinical routine diagnostics (Rossen et al., 2018). Therefore, there is an ongoing interest in rapid, cheap, accurate, and easy-to-use methods to identify bacteria isolated from the human intestinal tract.

Over the last decade, matrix-assisted laser desorption ionization time-of-flight mass spectrometry (MALDI-TOF MS) has been established as a technique for rapid and reliable bacterial identification and is mainly used in routine diagnostics (Bizzini and Greub, 2010; Li et al., 2019). The technique measures the mass spectral profiles (MSPs) within a range of 2000 to 20,000 Daltons, where also ribosomal proteins of bacteria occur. Usually, MSPs are compared within a commercial database to pre-recorded reference spectra and matching marker masses allow the identification of a species (Croxatto et al., 2012; Schumann and Maier, 2014; Seuylemezian et al., 2018). Quality of the spectra (Cuénod et al., 2021, 2022) and the database used are two critical factors for a reliable identification. The currently available commercial databases [we used the *MBT Compass reference library* (version 4.1.100)], which are widely used in routine diagnostics, only cover a minority of non-pathogenic *Clostridia*. Therefore, the current usage of MALDI-TOF MS for proper species identification is limited. This hurdle has been overcome in the past by researchers in other domains by the generation of in-house custom reference databases of bacterial strains relevant to their field of study (Calderaro et al., 2014; Fergusson et al., 2020; Moussa et al., 2021).

Considering the robust classification, rapid acquisition times, and low cost per sample of MALDI-TOF MS, we aimed to close this important gap and extend this technology to the identification of commensal members of the gastrointestinal microbiota of the class *Clostridia*. We generated 142 mass spectral profiles (MSPs) for 21 genera and 47 species, using full-length 16S rRNA gene sequence-confirmed bacterial isolates from diverse geographic regions. The newly created MSP library was evaluated for accuracy by re-identifying 58 blind-coded, full-length 16S rRNA gene sequence validated isolates of *Clostridia* covering the same genera and species using the new database plugin. Finally, we applied the new MSP library on a set of 326 isolates from stool samples of healthy Swiss donors, showing its potential on identifying new isolates from human fecal samples.

## Materials and methods

### Bacterial strains in the CLOSTRI-TOF database

The number and composition of the mass spectrum profile (MSP) library used reference library have a significant impact on the accuracy of the microflex Biotyper taxonomy assignment. To extend the coverage to the identification of members of non-pathogenic *Clostridia*, we used 142 sequenced strains. These strains are the main representatives of the human gut commensals of the class *Clostridia* (Table 1; Supplementary Table 1) and have been chosen to include as many geographically diverse strains as possible, to cover each species as broadly as possible. The 142 strains belonging to 21 genera and 47 species were confirmed for their species identification using full-length 16S rRNA gene sequencing. Fifty-eight (58) 16S rRNA gene full-length identified strains were used to validate the newly generated MALDI-TOF MS database. Strain information is summarized in Supplementary Table 1 (strains used for library construction), and Supplementary Table 2 (validation strains).

**Table 1:** Number of bacterial strains used to construct the CLOSTRI-TOF database.

Genus	Species	Total no. of strains included (no. of reference strains)
*Agathobacter*	*rectalis*	5 (0)
*Anaerobutyricum*	*hallii*	2 (1)
*Anaerostipes*	*hadrus*	5 (1)
*Anaerotruncus*	*colihominis*	5 (1)
*Blautia*	*caecimuris*	2 (1)
	*coccoides*	1 (1)
	*faecis*	5 (1)
	*glucerasea*	3 (0)
	*hansenii*	1 (0)
	*luti*	2 (1)
	*massiliensis*	4 (0)
	*obeum*	5 (1)
	*producta*	6 (2)
	*pseudococcoides*	1 (0)
	*schinkii*	5 (1)
	*wexlerae*	4 (1)
*Clostridium*	*leptum*	1 (1)
	*nexilis*	1 (0)
	*scindens*	5 (2)
	*symbiosum*	5 (1)
*Coprococcus*	*catus*	1 (1)
	*comes*	5 (1)
	*eutactus*	2 (0)
*Dorea*	*formicigenerans*	5 (1)
	*longicatena*	5 (1)
*Enterocloster*	*aldenensis*	3 (1)
	*clostridioformis*	5 (1)
*Eubacterium*	*ramulus*	2 (1)
*Faecalibacterium*	*prausnitzii*	4 (0)
	*longum*	1 (0)
*Faecalicatena*	*fissicatena*	5 (1)
*Fusicatenibacter*	*saccharivorans*	3 (1)
*Gemmiger*	*formicilis*	1 (0)
*Intestinibacter*	*bartlettii*	1 (1)
*Lachnospira*	*eligens*	3 (1)
*Lacrimispora*	*celerecrescens*	3 (1)
	*saccharolytica*	1 (1)
*Peptostreptococcus*	*stomatis*	1 (1)
*Roseburia*	*faecis*	2 (1)
	*hominis*	1 (0)
	*intestinalis*	1 (0)
	*inulinivorans*	2 (1)
*Ruminococcus*	*bromii*	3 (1)
	*gnavus*	4 (1)
	*lactaris*	3 (1)
	*torques*	2 (1)
*Sellimonas*	*intestinalis*	5 (1)

### Bacterial culture

The culture medium used for each strain is given in Supplementary Table 1 and included the following: Brain Heart Infusion with Inulin (BHII Agar): BHI Agar (BD Difco, cat. number 279830, Franklin Lakes, NJ, USA) supplemented with inulin from chicory (Sigma, cat. number I2255, Darmstadt, Germany), 1 g/l. Schaedler Agar (Oxoid, cat. number CM0497, Ireland), and modified Gifu Anaerobic Broth (mGAM) (HiMedia, cat. number M2079). All media were prepared according to manufacturer instructions and supplemented with 15 g agar/l whenever appropriate. Bacteria were cultured under strictly anaerobic conditions using an anaerobic workstation (Anaerobic chamber; Coy Laboratories, Ann Arbor, MI, USA) containing a gas mixture of 10% CO_2_, 5% H_2,_ and 85% N_2_. Bacteria were incubated at 37°C for 3–4 days before MALDI-TOF MS analysis. Culture conditions are summarized in Supplementary Table 1 (strains used for library construction) and Supplementary Table 2 (validation strains).

### DNA purification and 16S rRNA gene sequencing

Genomic DNA was extracted from bacterial cultures using a bead-beating device (SpeedMill BeadBeater for 30 s or with the vortex adapter on maximum speed for 10 min) followed by the Wizard genomic DNA purification kit (Promega, cat. number A2920, Dübendorf, Switzerland) according to manufacturer instructions. Total DNA was quantified by absorbance at 260 nm using the NanoDrop® ND-1000 Spectrophotometer (Witec AG, Littau, Switzerland). DNA samples were stored at-20°C until further analysis.

To confirm the identity of the isolates, the full region of the 16S rRNA gene was amplified by PCR using the FIREPol® DNA polymerase (Solis BioDyne, Tartu, Estonia) using primers 27F (5′-AGA GTT TGA TCC TGG CTC AG-3′) and 1492R (5′-GGT TAC CTT GTT ACG ACT T-3′) with the following conditions: 95°C for 3 min; 35 cycles of 95°C for 30 s, 50°C for 30 s, 72°C for 30 s; then 72°C for 7 min (Frank et al., 2008). PCR products were visualized using standard gel electrophoresis (1% agarose in 1× Tris-acetate-EDTA [TAE] buffer) at 100 V for 30 min (Bio-Rad, Cressier, Switzerland), purified using the Promega Wizard™ SV Gel, and PCR Cleanup System according to the manufacturer’s protocol and sequenced bi-directionally to obtain near full-length 16S rRNA gene sequence using Sanger sequencing at Microsynth (Balgach, Switzerland). To identify the closest homologs, the obtained sequences were aligned using the Basic Local Alignment Search Tool (BLAST) (Altschul et al., 1990). We defined sequence homology ≥99% as a species match, and sequence similarity ≥97% as a genus match (Becker et al., 2004). To build the phylogenetic tree, 16S rRNA gene sequences were aligned and trimmed using MUSCLE and a maximum-likelihood phylogenetic tree was constructed with 1,000 bootstrap replicates using MEGA 11 (Tamura et al., 2021).

### MALDI-TOF MS spectra preparation for MSP creation

As recommended by Bruker-Daltonics for new library creations, ethanol-formic acid extraction was used to prepare the sample for MSP creation. Formic acid is routinely used when using MALDI-TOF in bacteriology since acidic pH of the matrix improves the extraction of the ribosomal proteins (Croxatto et al., 2012). Briefly, two to three colonies were suspended in 300 μl of high-pressure liquid chromatography (HPLC)-grade water (Sigma-Aldrich, Buchs, Switzerland) and mixed with 900 μl of 100% ethanol (Sigma-Aldrich, Buchs, Switzerland). After centrifugation at 15,000× *g* for 2 min, the supernatant was removed, and the pellet was dried. For sample extraction, 50 μl of formic acid (70% in water) was added to the bacterial pellet, the tube was thoroughly mixed by vortexing and 50 μl acetonitrile (Sigma) were added to the mixture. The mixture was centrifuged at 15,000× *g* for 2 min. For each strain, 1 μl of the supernatant containing the bacterial extract was spotted on a 96-spot steel plate (Bruker-Daltonics) in 15 replicates and allowed to dry at room temperature. One microliter of bacterial test standard (BTS, Bruker-Daltonics) was pipetted on two MALDI target spots for each plate to allow for calibration during acquisition and processing. Subsequently, the samples were overlaid with 1 μl of α-cyano-4-hydroxycinnamic acid (HCCA) matrix and air dried again prior to measurement. A Bacterial Test Standard (BTS) was used to calibrate the instrument, before each acquisition session.

### MALDI-TOF MS data acquisition

MALDI-TOF MS was performed with a Bruker-Daltonics microflex LT/LH bench-top mass spectrometer. Protein mass spectra of samples were acquired in a mass range of 2000–20,000 Da using the flexControl 3.4 program (Bruker-Daltonics) and a laser frequency at 20 Hz with a linear positive mode. The default operating conditions were as follows: ion source 1, 20 kV; ion source 2, 18.25 kV; pulse ion extraction, 370 ns. Each spectrum was summed up to 240 laser shots (40 laser shots/position x 6 different positions). For each strain, 30 single spectra were generated from two independent cultures with 15 technical replicates. All raw spectra have been deposited on Zenodo with the access number 10.5281/zenodo.7573939.

### MALDI-TOF MS quality control and custom MSP library creation

**Figure 1:**
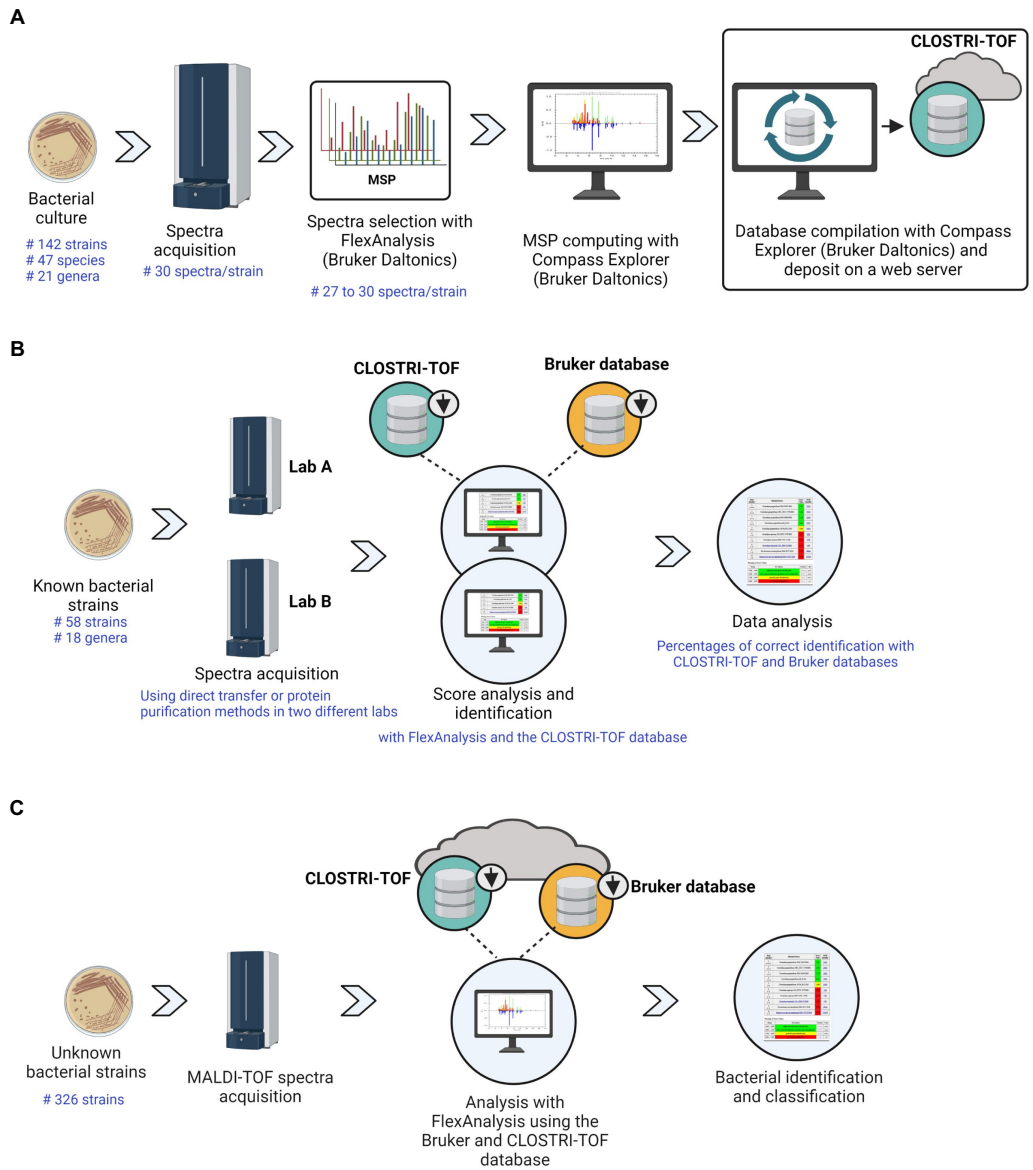
Main steps of CLOSTRI-TOF creation **(A)**, validation **(B)**, and MALDI-TOF MS microbial identification workflow **(C)**.

The quality of raw spectra was evaluated using flexAnalysis 3.4 software (Bruker-Daltonics). To ease the quality check of the raw spectra, smoothing parameters and baseline subtraction parameters were applied. For baseline subtraction we used the multipolygon, minimum, penalized least squares, and penalized B-Spline baseline subtraction method, and for smoothing, we used the Savitzky-Golay smoothing method. For each analysis day, the eight known masses of the bacterial test standard (BTS) spectrum were controlled to be within the tolerance range of +/− 300 ppm to prove mass accuracy. Spectra showing any flatline, outliers, dramatic mass shifts, and anomalies were deleted. Flatline spectra were defined as spectra with a completely flat line and outliners were defined as spectra with a unique peak that was detected in no other spectra of the same strain. Mass shifts were assessed on a random peak between 6,000 Da to 7,000 Da and we only accepted spectra with peak shifts below 500 ppm. After processing the raw spectra, a minimum of 27 high-quality spectra per strain were selected and merged using the MALDI Biotyper Compass Explorer software (Bruker-Daltonics) to create the mass spectral profiles (MSPs). The merged spectra per strain constituting the MSP were verified to have a log score greater than 2.7 and a peak frequency greater than 75%. The final library was compiled based on the MSP of 142 strains (Figure 1A). An MSP dendrogram was constructed using a Euclidean distance measure and an average linkage algorithm with the MALDI Biotyper Compass Explorer 4.1. The final library has been deposited on Zenodo with the access number 10.5281/zenodo.7573939.

### CLOSTRI-TOF database validation

To validate the CLOSTRI-TOF database, we performed a blind test of 58 full-length 16S rRNA gene sequenced isolates in two collaborating laboratories (Figure 1B; Supplementary Table 2). Validation was performed using the extended direct transfer (eDT) technique. Briefly, a few bacterial colonies were directly smeared onto a MALDI-TOF MS target and covered with a 1 μl of saturated HCCA matrix solution as is generally recommended for routine bacterial identification. In case no spectra were detected, we repeated the analysis using the ethanol-formic acid extraction method (as previously described). The raw spectra generated were compared to the two combined databases (Bruker-Daltonics MALDI Biotyper Compass MSP library v. 4.1.100 and CLOSTRI-TOF) (Figure 1B). The identification results were classified using the score values proposed by Bruker: the highest matching scores of the spectra were represented by ranges indicating high confidence identification (2.000–3.000), low confidence identification (1.700–1.999) and no organism identification possible (0.000–1.699).

### Identification of a set of 326 clinical isolates from healthy Swiss volunteers

To test the library on a set of previously unknown isolates, we used the CLOSTRI-TOF database combined with the original Bruker-Daltonics MALDI Biotyper Compass MSP library v. 4.1.100 to identify a total of 326 anaerobic cultures isolated from stool samples of healthy Swiss individuals recruited in the EnrichBut project (Swiss Ethics Number: BASEC ID 2021-00199) (Figure 1C). In brief, stool samples were collected from four self-declared healthy Swiss adults between 30 and 60 years of age who did not consume antibiotics two weeks prior to sample collection. Each stool donor was provided with a custom-made stool sampling kit that allows for the preservation of the oxygen sensitive bacterial groups under anaerobic conditions. Stool samples were stored anaerobically at room temperature prior to processing. Upon receipt (within 48 h of collection), the stool samples were resuspended (1:10 w/v) in anaerobic phosphate-buffered saline (PBS). The suspensions were then serially diluted in PBS and 100 μL was plated onto Columbia Blood, BHII, Schaedler and mGAM Agar. The plates were incubated at 37°C for 5 days. Between 20 and 30 colonies showing different colony morphologies were picked from each medium and re-streaked three times on fresh agar plates to ensure purity of the isolates. The isolates were cultured in liquid broth and mixed with 20% glycerol and stored at – 80°C.

The isolates were prepared for MALDI-TOF MS identification using the extended direct transfer (eDT) technique and were identified based on three technical replicates. The obtained spectra were compared either against the commercial Bruker-Daltonics library or the combined library from Bruker-Daltonics and CLOSTRI-TOF.

## Results

### MSP library creation

For the 142 strains analyzed, we generated 2,130 target positions and a total of 4,260 individual mass spectra. The quality of the spectra was high, with an average of 1–3 of the raw spectra removed per strain prior to MSP generation. Thus, we could generate a robust library for the 47 species.

### Evaluation of MSP library performance

**Figure 2:**
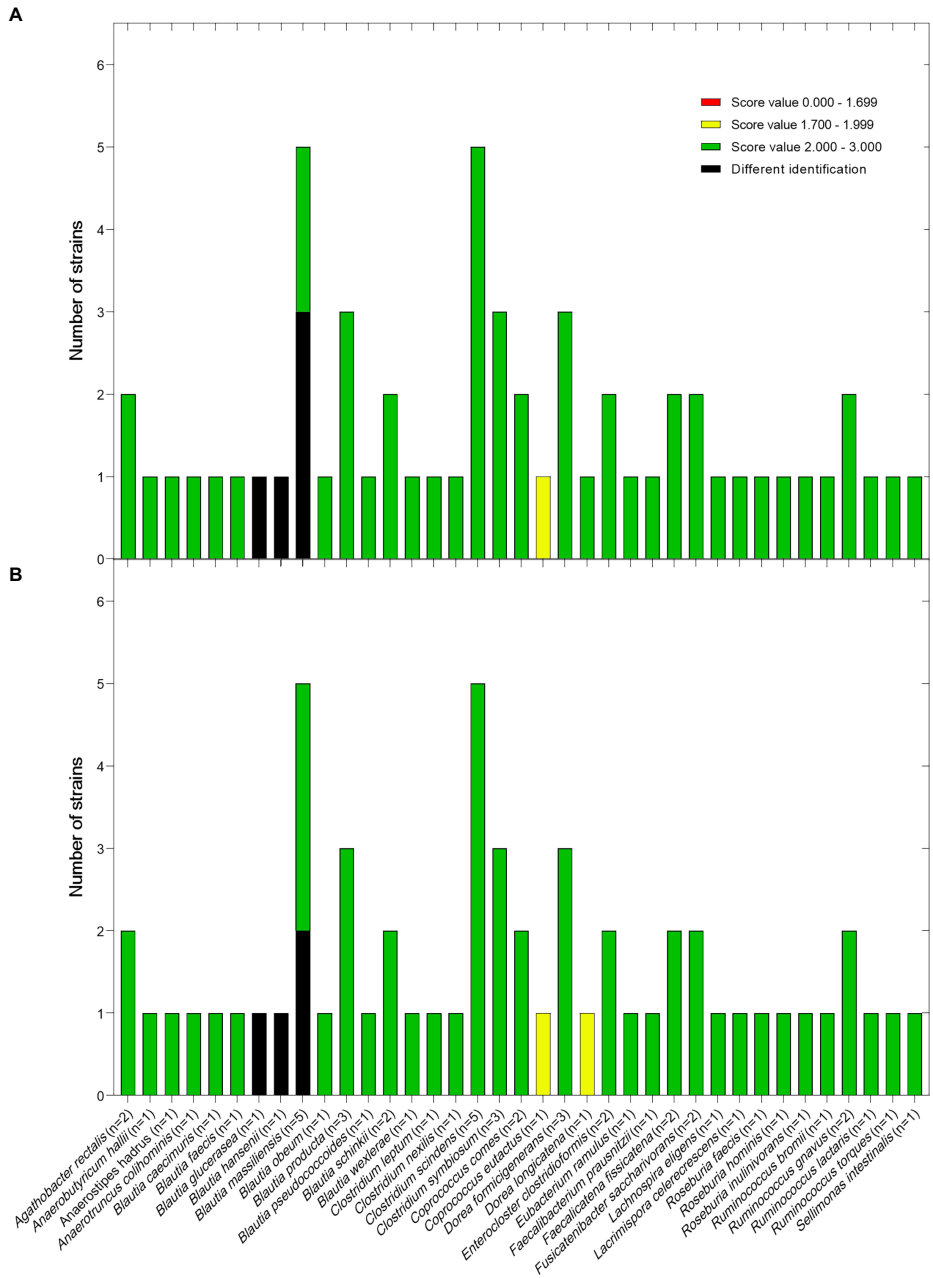
Blind test of *Clostridia* strains using the newly created CLOSTRI-TOF MS performed in lab A **(A)** and lab B **(B)**. The highest matching scores of the spectra were represented by ranges indicating high confidence identification (2.000–3.000), low confidence identification (1.700–1.999) and no organism identification possible (0.000–1.699).

To test the newly established library, we subjected 58 additional strains (validations strains, Supplementary Table S2), not included in the set of strains used for library construction but previously identified based on their 16S rRNA gene sequences to MALDI-TOF MS. Combining the original Bruker database with the CLOSTRI-TOF database plugin allowed reliable identification of at the species level (score value ≥2.0) of 57/58 strains (98.3%) in laboratory A and 54/58 (93.1%) in laboratory B (Figures 2). Of note, several strains belonging to the genus *Blautia* were identified with a high score (≥2.0) but were incorrectly assigned at the species level. Further, *Coprococcus eutactus* was correctly identified in both laboratories, but with a score between 1.7 and 2.0. All the validation strains (58/58) (100%) were reliably identified at the genus level (score ≥ 1.7 but <2.0) in both laboratory A and B. Thus, using the combination of the original Bruker database and the CLOSTRI-TOF database, we were able to reidentify most of the validation strains to species level and all strains to genus level.

### 16S rRNA gene-based phylogeny and MALDI-TOF MS profiling

**Figure 3:**
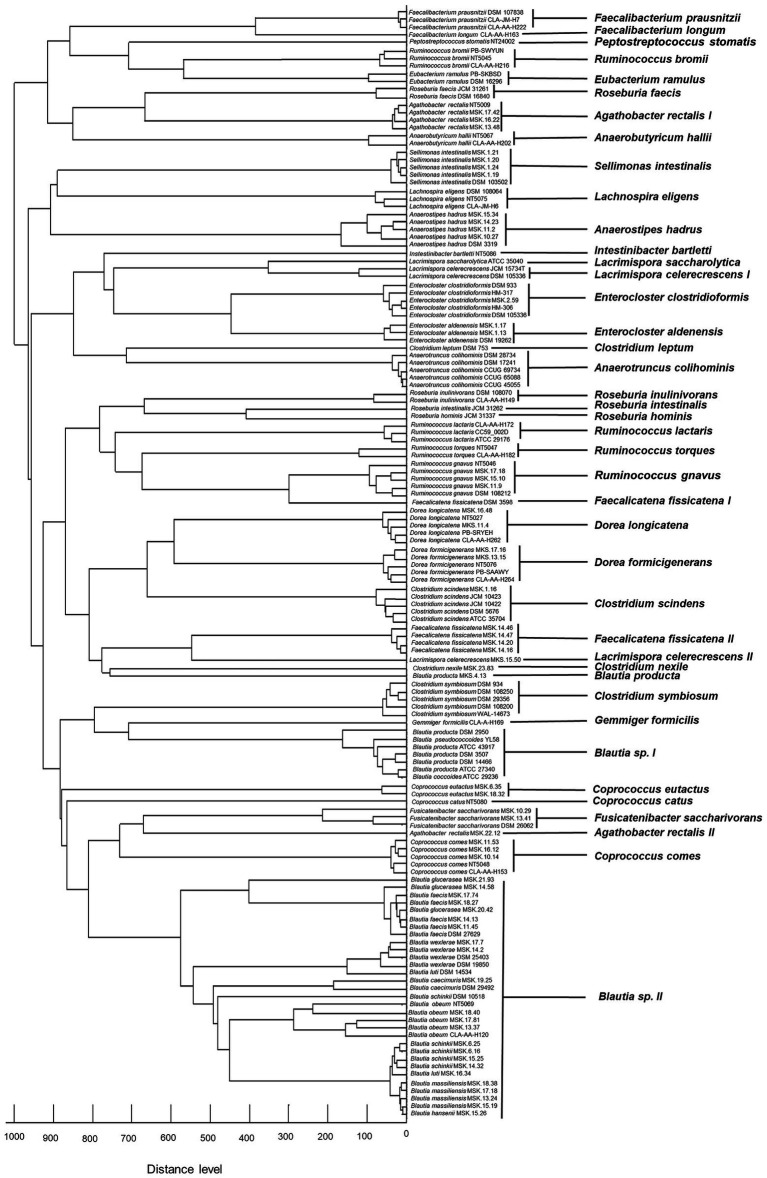
Distribution of different species with similar spectral profiles according to MSP dendrogram cluster analysis.

**Figure 4:**
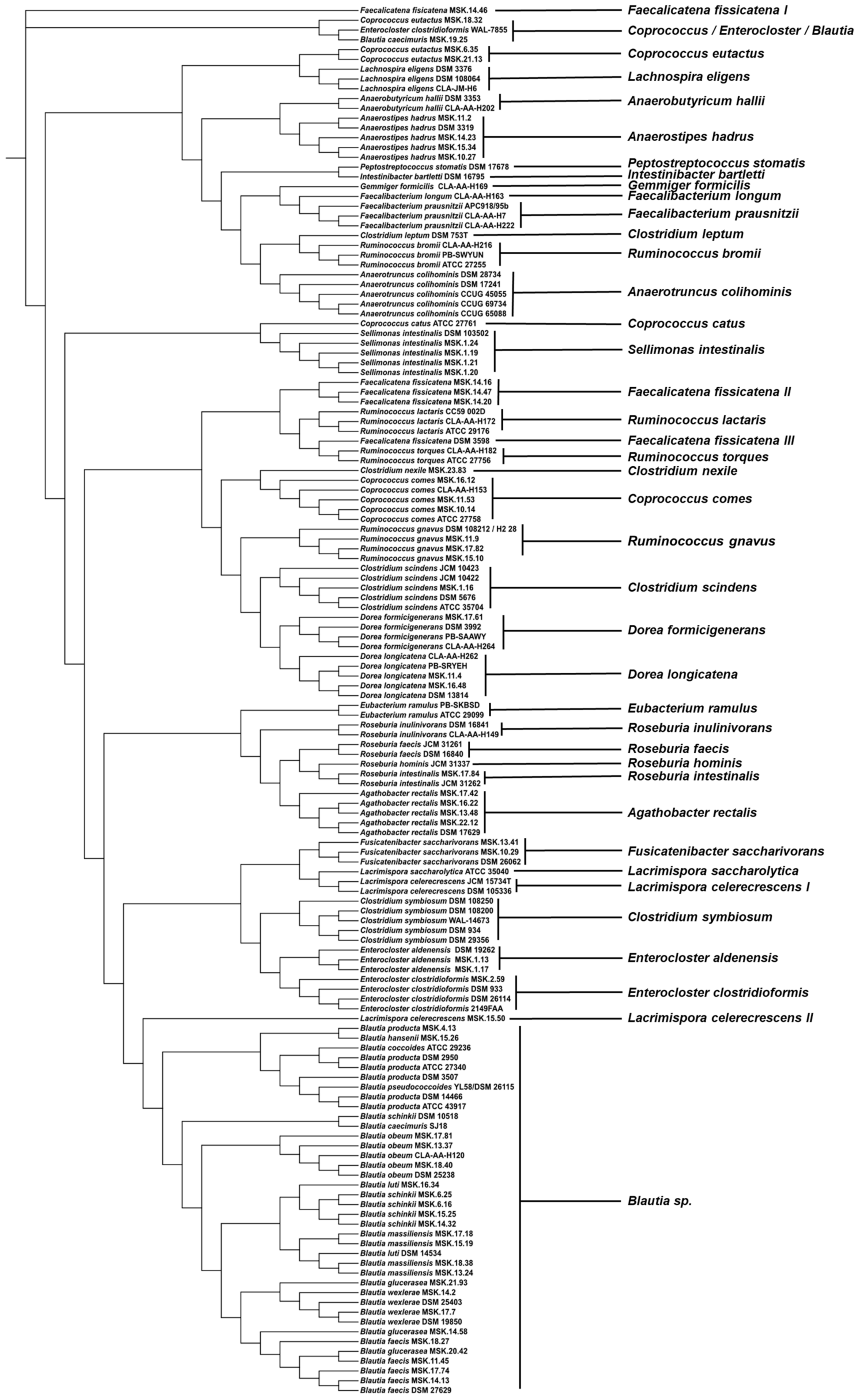
Maximum-likelihood phylogenetic tree based on complete 16S rRNA gene sequences of the strains used in the construction of CLOSTRI-TOF database.

To investigate the taxonomic assignment relationship between the microflex Biotyper and 16S rRNA gene-based sequencing, we compared dendrograms generated by either of these techniques. In most cases, the two methods provided identical results where strains belonging to the same species clustered together (Figures 3, 4). However, different strains of *Agathobacter rectalis*, *Blautia* spp., *Faecalicatena fissicatena,* and *Lacrimispora celerecrescens* formed two or three distinct MALDI-TOF-based clusters (Figure 3). Likewise, the maximum-likelihood phylogenetic tree based on 16S rRNA gene sequences was unable to cluster together strains of the same species of *Agathobacter rectalis*, *Blautia* spp., *Faecalicatena fissicatena,* and *Lacrimispora celerecrescens* (Figure 4).

### Identification of anaerobic gut bacterial isolates using MALDI-TOF MS enhanced by CLOSTRI-TOF

**Figure 5:**
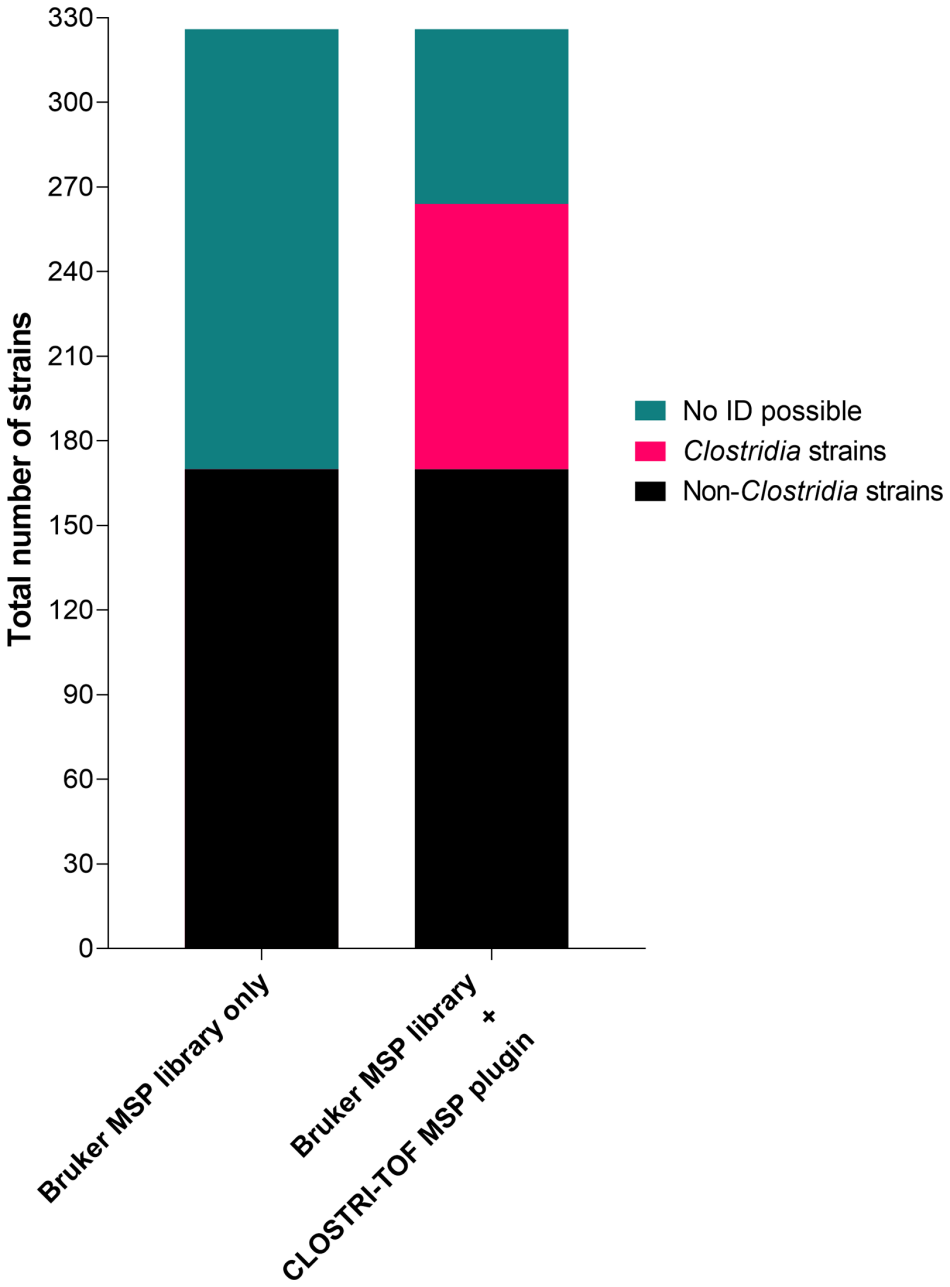
Identification rates for 326 bacteria isolates tested using the Bruker-Daltonics MALDI Biotyper Compass MSP library v. 4.1.100 combined or not with the CLOSTRI-TOF database plugin.

We next analyzed a bank of 326 strains isolated from the feces of healthy Swiss volunteers (Supplementary Table 5) using the microflex Biotyper. Of the 326 analyzed strains, 170 (52.1%) were identified with the original Bruker database, while 156 (47.9%) could not be identified/had no match in the library at a score > 1.7. Combining the original library with the CLOSTRI-TOF library plugin, we increased the number of strains identified at a score > 2.0 to 264 (80.9%) (Figure 5), which corresponds to an identification of formerly unknown strains of 60%.

## Discussion

To date, 16S rRNA gene sequencing is one of the most widely used techniques to identify unknown microbial isolates from the human gastrointestinal tract. Here, we propose a MALDI-TOF MS library plugin, CLOSTRI-TOF, enhancing the power of MALDI biotyping as a rapid, accurate, and economical alternative.

MALDI biotyping is strongly dependent on spectral databases, thus allowing to identify only taxa that already have spectral information for a given species. This hurdle has been overcome by the generation of in-house custom reference databases for specific groups of microorganisms. As an example, previous research has generated database plugins for different species of the genera *Borrelia* (Calderaro et al., 2014)*, Burkholderia* (Fergusson et al., 2020) or *Vibrio* (Moussa et al., 2021), different strains of *Clostridium tyrobutyricum* (Burtscher et al., 2020) as well as for helminths (Sy et al., 2022).

The CLOSTRI-TOF library plugin presented here allowed to identify all the 47 bacterial species included in the library plugin at the genus level and most even down to species level. Further, we could show that the combination of the original Bruker library combined with the CLOSTRI-TOF library plugin significantly increase the number of identified strains from a collection of fecal bacterial isolates of healthy Swiss volunteers. One of the species included in our library (*Coprococcus eutactus*) did not yield reliable identification using the validation strains. Of note, *C. eutactus* is represented by only two strains in the CLOSTRI-TOF library. It has been shown previously that the number of included strains in the database is critical to account for intraspecific diversity, thus allowing reliable identification (Erler et al., 2015). Future updates to the CLOSTRI-TOF database should therefore expand the number of strains included for species with a low strain coverage.

While our database plugin allowed identification of *Blautia* at the genus level, it did not allow reliable assignment at species-level. As the strains of *Blautia* were also polyphyletic in the 16S rRNA gene phylogenetic tree, it would be interesting to compare the phylogenetic relationship within this genus in more detail. The library plugin presented here is focused on the main members of the class *Clostridia* colonizing the human gastrointestinal tract. We have chosen this group of microorganisms, as they are frequently involved in the production of the key metabolite butyrate (Louis and Flint, 2009) and reduced in dysbiotic diseases, including undernutrition (Vonaesch et al., 2018), ulcerative colitis (Machiels et al., 2014), and type 2 diabetes (Wang et al., 2012). Expanding the database beyond the class of *Clostridia* to other frequent members of the human microbiome will be key for rapid identification of all bacterial strains isolated from human fecal samples.

Of note, MALDI biotyping allows for rapid identification of bacteria, yet is not able to give the same amount of information on each isolate as would be gained by whole-genome sequencing. Thus, MALDI identification should be seen as a low cost, rapid pre-identification tool to select the strains that will be characterized in greater detail using more advanced methods such as whole-genome sequencing.

## Conclusion

Our data show that MALDI-TOF MS is a promising tool to rapidly identify isolates of commensal bacteria. The new open-source library plugin developed here allows to discriminate all taxa included to the genus and most taxa even down to species level.

## Data availability statement

The datasets presented in this study can be found on Zenodo under the following link: https://zenodo.org/record/7773644#.ZCny2y8Rrfc.

## Author contributions

PA, LM, and PV: conceptualization. VT, TW, GG, TC, EP, and AE: resources. PA, CL, VH, YT, AC, NA, CG, AA, MC, CK, and PV: methodology. PA, CL, and PV: data curation, formal analysis, visualization, and writing of original draft. PA, CL, GG, TC, EP, AE, LM, and PV: review and editing. All authors contributed to the article and approved the submitted version.

## Funding

This work was funded by the Bill and Melinda Gates Foundation (grant number INV-004352 to PV and LM), the Forschungsfonds of the University of Basel (PV) and an Eccellenza Fellowship from the Swiss National Science Foundation to PV (grant number PCEFP3_194545). The Vonaesch and Greub labs are part of NCCR Microbiomes, a National Centre of Competence in Research, funded by the Swiss National Science Foundation (grant number 180575). TC received funding from the German Research Foundation (DFG), project ID 403224013—SFB 1382, project ID 395357507—SFB 1371, and project ID 460129525—NFDI4Microbiota. NA, CG, and LM are supported by DFG (CMFI Cluster of Excellence EXC 2124 and Emmy Noether Program).

## Conflict of interest

TW and VH were employed by PharmaBiome AG.

The remaining authors declare that the research was conducted in the absence of any commercial or financial relationships that could be construed as a potential conflict of interest.

## Publisher’s note

All claims expressed in this article are solely those of the authors and do not necessarily represent those of their affiliated organizations, or those of the publisher, the editors and the reviewers. Any product that may be evaluated in this article, or claim that may be made by its manufacturer, is not guaranteed or endorsed by the publisher.
