# Corrigendum: A MALDI-TOF MS library for rapid identification of human commensal gut bacteria from the class *Clostridia*

**DOI:** 10.3389/fmicb.2023.1208177

**Published:** 2023-05-22

**Authors:** Paul Tetteh Asare, Chi-Hsien Lee, Vera Hürlimann, Youzheng Teo, Aline Cuénod, Nermin Akduman, Cordula Gekeler, Afrizal Afrizal, Myriam Corthesy, Claire Kohout, Vincent Thomas, Tomas de Wouters, Gilbert Greub, Thomas Clavel, Eric G. Pamer, Adrian Egli, Lisa Maier, Pascale Vonaesch

**Affiliations:** ^1^Department of Fundamental Microbiology, University of Lausanne, Lausanne, Switzerland; ^2^Department of Epidemiology and Public Health, Swiss Tropical and Public Health Institute, Basel, Switzerland; ^3^University of Basel, Basel, Switzerland; ^4^Applied Microbiology Research, Department of Biomedicine, University of Basel, Basel, Switzerland; ^5^Clinical Bacteriology and Mycology, University Hospital of Basel, Basel, Switzerland; ^6^Interfaculty Institute of Microbiology and Infection Medicine Tübingen, University of Tübingen, Tübingen, Germany; ^7^Cluster of Excellence ‘Controlling Microbes to Fight Infections', University of Tübingen, Tübingen, Germany; ^8^Functional Microbiome Research Group, Institute of Medical Microbiology, RWTH University Hospital, Aachen, Germany; ^9^Institute of Microbiology of the University of Lausanne, University Hospital Centre (CHUV), Lausanne, Switzerland; ^10^Duchossois Family Institute, Division of Infectious Diseases and Global Health, University of Chicago, Chicago, IL, United States; ^11^BioAster, Paris, France; ^12^PharmaBiome AG, Schlieren, Switzerland

**Keywords:** human gut microbiota, *Clostridia*, MALDI-TOF MS, commensal bacteria, anaerobic, bacterial identification, culturomics, next-generation probiotics

In the original article, there was an error in the **Data availability statement**. The dataset deposited on the Zenodo link was incomplete and the MALDI spectra were named with the old taxonomy. We have now corrected these errors, added a few strains and taken out two strains with ambiguous spectra. We have uploaded the ClostriTof version 2.0 database plugin on Zenodo. The correct **Data availability statement** appears below.

“The datasets presented in this study can be found on Zenodo under the following link: https://zenodo.org/record/7773644#.ZCny2y8Rrfc.”

In the published article, there was an imprecision in the **Conflict of interest**. This sentence previously stated: “The authors declare that the research was conducted in the absence of any commercial or financial relationships that could be construed as a potential conflict of interest.” The correct **Conflict of interest** appears below.

“TW and VH were employed by PharmaBiome AG.

The remaining authors declare that the research was conducted in the absence of any commercial or financial relationships that could be construed as a potential conflict of interest.”

The authors apologize for these errors and state that this does not change the scientific conclusions of the article in any way. The original article has been updated.

